# Dry matter and crude protein degradability of Napier grass (*Pennisetum purpureum*) silage is affected by fertilization with cow-dung bio-digester slurry and fermentable carbohydrate additives at ensiling

**DOI:** 10.1093/tas/txac075

**Published:** 2022-06-23

**Authors:** Mashudu D Rambau, Felix Fushai, Todd R Callaway, Joseph J Baloyi

**Affiliations:** Department of Animal Science, University of Venda, Thohoyandou, Limpopo, South Africa; National Agricultural Marketing Council, Pretoria, Gauteng, South Africa; Department of Animal Science, University of Venda, Thohoyandou, Limpopo, South Africa; Department of Animal and Dairy Science, University of Georgia, Athens, GA 30602, USA; Department of Animal Science, University of Venda, Thohoyandou, Limpopo, South Africa

**Keywords:** biodigester, carbohydrate additives, Napier grass (*Pennisetum purpureum*), silage degradability

## Abstract

Dry seasons pose a major nutritional constraint on ruminant livestock production in tropical regions, which justifies forage conservation to meet the dry season feed requirement. Napier grass is a tropical forage that is used for silage in South Africa. The present objective was to determine the effects of Napier grass fertilization with bio-digester slurry (BDS) and the inclusion of fermentable carbohydrate additives at ensiling on the chemical composition and ruminal degradability of Napier grass silage. Napier grass was established in 5 × 4 m plots, replicated three times in a completely randomized design, and irrigated weekly with either BDS or water. After 12 weeks, the Napier was cut and ensiled for 90 days in 1-liter glass jars in a 2 (BDS, water) × 4 (no-additive, molasses, brown sugar, and maize meal) factorial arrangement replicated three times. The nutrient composition was determined using standard protocols. The ruminal degradability of dry matter (DM) and crude protein (CP) was determined using the nylon bag technique. Fertilization with BDS increased (*P* < 0.05) pH and CP and reduced (*P* < 0.05) fat content of fresh-cut Napier. Additives increased (*P* < 0.01) silage DM content and reduced (*P* < 0.01) acid detergent fiber, neutral detergent fiber content. The BDS fertilization with molasses inclusion increased (*P* < 0.05) silage DM relative to the no-additive and maize meal inclusion, and decreased (*P* < 0.05) fat content compared to the no-fertilizer, added maize meal silage. Molasses increased silage water-soluble carbohydrate and decreased the NH_3_-N content (*P* < 0.05) compared to the no-additive and maize meal treatments. For DM, the BDS fertilized, no additive silage had the least “a” fraction (*P* < 0.01), while the no BDS, no-additive silage had the least “b” fraction (*P* < 0.01), with least (*P* < 0.01) potential degradability (PD) observed for the no BDS, no-additive treatment. Fertilization increased (*P* < 0.01) effective degradability of DM at outflow rates *k* = 0.02, 0.05, 0.08, with same effect for molasses and maize meal inclusion. Relative to the control, molasses inclusion increased (*P* < 0.01) PD of silage CP. In conclusion, our results suggested BDS fertilization of Napier grass ensiling with added readily fermentable carbohydrate substrate, particularly from molasses, induced changes in silage chemical and fermentation characteristics likely to promote better forage preservation and ruminal microbial function.

## INTRODUCTION

In tropical environments, dry seasons pose a major nutritional constraint on ruminant livestock production. Cultivation and ensilage of surplus forages during rainy seasons can mitigate the forage deficit. Generally, ensiling is considered as an efficient process of preserving forage with high moisture content in sufficiently good quality. Napier grass (*Pennisetum purpureum*) is a native grass grown widely in Southern Africa that is commonly used as a silage crop in tropical climates due to its high quality and yield (Bureenok et al., 2012). However, the quality of Napier grass silage depends on that of the harvested forage quality and composition (Loures et al., 2003).

To achieve high yields of quality forage, the maintenance of soil fertility is critical. Organic soil amendments are often applied to increase crop productivity, crop quality, or both (Edmeades, 2003). Bio-digester slurry (BDS) is the by-product of gas production generated from bio-degradable products through anaerobic degradation. While the BDS contains substantial, amounts of nitrogen (N), phosphorus, and potassium which are recommended to promote soil health for sustainable cropping systems, the nutritional benefits of the slurry irrigation are not clearly defined for specific pasture species (Gurung, 1997).

Effective ensilage can reduce the cost of feeding ruminants and ensure a steady supply of quality feed (Pirmohammadi et al., 2006). However, tropical grasses contain high crude protein (CP) content, and characteristically low fermentable carbohydrates compared to forage maize (Markos and Fulpagare, 2015), attributes which may reduce the silage quality (Nisa, 2006).

Unfortunately, Napier grass contains low levels of highly fermentable, water-soluble carbohydrates (WSCs), which can be increased through application of carbohydrate additives to enhance silage quality and increase animal productivity (Tauqir et al., 2009). Readily available, low-cost carbohydrate additives such as brown sugar, molasses, and maize meal can be used to improve the quality of silage produced by poorly resourced farmers. Therefore, the aim of the study was to determine the effects of irrigation of Napier grass with BDS and of inclusion of carbohydrate additives at the time of ensiling on the chemical quality and ruminal degradability of Napier grass silage.

## MATERIALS AND METHODS

### Experimental Site

The study was conducted in South Africa at the University of Venda, School of Agriculture Experimental Farm (22°58ʹ32″ S, 30°26ʹ45″ E; Altitude of 596 m above sea level). The area receives annual rainfall of ±500 mm that falls predominantly in summer. The average annual maximum and minimum temperatures are 31 °C and 18 °C, respectively. The area is characterized by deep, well drained red clay soils with low organic carbon, and has a slightly acidic pH.

### Ethical Clearance

The experimental procedure was approved by the Ethics Committee of the University of Venda (SARDF/16/ANS/05).

### Napier Grass Production and Experimental Design

Napier grass was initially planted by ploughing using a tractor, harrowing, marking. The prepared land was demarcated into six (6) 4 × 5 m plots to which two (2) fertilization treatments were allocated in a completely randomized design (CRD) replicated three times. Napier cuttings with three nodes were planted manually, two nodes in the ground and one up, at an angle of 30–45°, spaced 70 cm inter, and intra-rows. In the second season after planting, the Napier grass was harvested by cutting about 15 cm above the ground (Mtengeti et al., 2006) to allow uniform regrowth prior to the harvest.

Napier fodder was manually supplementary irrigated weekly with either BDS or water, both at 30 m^3^ ha^−1^, using 10-liter (ℓ) watering cans. The BDS slurry was from a bio-digester fed cattle dung and water in a 1:1 ratio every day. Bulk BDS was then further diluted with water at a ratio of 1:1 for easier application. Plots were kept weed-free for the 12-week experimental period by hand hoeing, after which the Napier fodder was hand harvested back to 15 cm above ground and the harvested forage machete-chopped to approximately 1.2–1.27 cm length. The effect on Napier fodder quality of the irrigation treatments (Table 1) was evaluated on approximately 1,000 g of fresh Napier grass samples collected randomly from each plot pre-ensiling.

**Table 1.** Dry matter (g kg^−1^), mineral composition (g kg^−1^ DM), and pH of the experimental cattle bio-digester slurry

Component	Concentration
Dry matter	12.11
pH	8.11
Calcium	0.18
Magnesium	0.28
Nitrogen	0.35
Phosphorus	0.02
Potassium	0.88
Sodium	0.20

### Ensiling and Experimental Design

For each fertilization treatment, samples of chopped forage separately harvested from each of triplicate replicate plots for ensiling were weighed (approximately 600 g wet basis), and four carbohydrate additives (no-additive [control], molasses, brown sugar, and maize meal) were spread at 10% *(w/w)* of the total wet weight. To be able to flow better for an even spread on the chopped material, the molasses was pre-thinned by sun-heating within a container to a sufficiently fluid consistency. Additives with the forage were mixed thoroughly and ensiled in 1 ℓ Consol anaerobic bottle jars, and each treatment combination was replicated three times. The grass was compressed using pruning scissors to squeeze air out of the jars, to promote anaerobiosis. The jars were tightly sealed with lids that were preheated in warm water, sellotaped and then stored at room temperature for 90 days. The experiment was designed as a 2 (fertilization treatment) × 4 (carbohydrate additives) factorial arrangement.

### Analysis of Fresh Cut and Ensiled Napier Grass

Fresh-cut grass and silage samples obtained after 90 days of fermentation were analyzed in the Animal Science Nutrition Laboratory, University of Venda, Thohoyandou. A pH meter (Accumet AB150 pH/mV; Fisher Scientific; Singapore) was used to measure the pH according to Mtengeti et al. (2006). The samples were dried at 60 °C in an oven for 48 h to determine dry matter (DM) content (AOAC, 1990) and ground through a 1 mm screen size. The WSC content was determined using the Anthrone method (Murphy, 1958). Ash was analyzed by combusting at 550 °C overnight (AOAC, 1990). The N content was determined using the Kjeldahl procedure (AOAC, 1990) and the CP was calculated as N × 6.25. Non-protein Nitrogen (NPN) was determined according to Licitra et al. (1996). Fat content was determined using the Soxhlet fat extraction method (AOAC, 1990). Forage neutral detergent fiber (NDF), acid detergent fiber (ADF), and acid detergent lignin (ADL) contents were determined using the technique of van Soest et al. (1991). After 90-day of ensiling, in addition to nutrient analyses described for the fresh forage, the silage was analyzed for Lactic acid (LA) according to Faithfull (2002), and for Ammonia Nitrogen (NH_3_-N) (AOAC, 1990; method 941.04).

### Ruminal Degradability

Three mature Bonsmara steers, surgically fitted with rumen cannulae of 10 cm center diameter purchased from Bar Diamond Inc. were used to determine the degradability profiles of DM and CP of the Napier grass silage. The animals were housed in open, shaded feedlot pens and fed a 120g/kg DM CP commercial complete cattle finisher diet ad libitum, starting 21 days prior to the commencement of the ruminal incubation of nylon bags. Clean drinking water was available at all times in water troughs.

Representative silage from each treatment combination were oven dried at 60 °C for 48 h and ground to pass a grinding mill of 1 mm screen size before incubation in the rumen.

The nylon bag technique of Ørskov and McDonald (1979) was used. Representative silage samples composited across replicated plots within each fertilization × additives treatment combination of approximately 5 g each were weighed in well-labeled nylon bags (external dimension: 6 × 12 cm, pore sizes of 46 µm). The sample bags were duplicated within each animal per incubation period in the rumen giving a total of 384 samples. The sealed nylon bags were attached using plastic bands to flexible vinyl plastic tubes (40 cm long × 6 mm outer diameter) resistant to rumen microbial fermentation which were tied with 10 cm plastic ropes secured to a rubber stopper for continuous suspension in ruminal fluid. Sample nylon bags were inserted in the rumen at 06:00 h, immediately before the morning feeding time.

The bags were subsequently withdrawn after 0, 6, 12, 24, 48, 72, 96, and 120 h rumen incubation times and were immediately washed under low running tap water while rubbing gently between thumb and finger, till the water ran clear and were rinsed with deionized water. The zero-hour (control) bags were washed similarly without incubation in the rumen. Washed bags were dried in a forced-air oven at 60 °C for 48 h (AOAC, 1990), desiccated for 30 min, and then weighed to determine DM content. The final residues in all bags were composited by the silage treatment, incubation hour and steers and subsequently ground through a 1 mm sieve and analyzed in duplicate. Residues were analyzed for N content using the Kjeldahl procedure (AOAC, 1990) and N was converted to CP using the formula of N% × 6.25. Protein and DM nutrient degradation constants at each time for each sample were estimated using the Neway “Fitcurve” Excel software version 6, which was computed using mathematical the model of Ørskov and McDonald (1979):


$$\begin{array}{l} \;P=a+b(1-e^{-ct}) \end{array}$$

where, *P* = the DM disappearance at time *t*; *a* = the zero-time intercept (soluble fraction); *b* = the slowly degradable fraction; and *c* = the rate of degradation.

Potential degradability (PD) of DM and CP was estimated as (*a* + *b*), and the effective degradability (ED) was calculated using rumen fractional outflow rates (*k*) of 0.02, 0.05, and 0.08 per h according to Ørskov and McDonald (1979):


$$\begin{array}{l} ED=a+\frac{bc}{\left(k\;+c\right)} \end{array}$$

### Statistical Analysis

Analyses of variance on fresh-cut grass (Model I), silage quality (Model II), and degradability chemical composition data (Model III) were performed using the General Linear Model procedures of Minitab Statistical package version 17 (Minitab Inc., State College, PA).


$$\begin{array}{l} \;\;\;Y_{ijk}=\mu +S_{i\;}+C_{j}+\;{\left(SC\right)}_{ij}+\;\varepsilon_{ijk}\;Model\;\;\;I \end{array}$$

where, *Y*_*ij*_ = the observation—pH, DM, WSC, CP, NDF, ADF, ADL, Fat, Ash, minerals, *µ* = overall mean common to all observations; *S*_*i*_ = effect of *i*th BDS, *i* = 1 or 2 and *Ԑ*_*ij*_ = random residual error.


$$\begin{array}{l} Y_{ijkl}=\mu +A_{i\;}+S_{i}+C_{jk}+{\left(SC\right)}_{ij}+\;\varepsilon_{ijkl}\;Model\;II \end{array}$$

where, *Y*_*ijk*_ = the observation—pH, DM, WSC, CP, NDF, ADF, ADL, Fat, Ash, minerals, *µ* = overall mean common to all observations; *S*_*i*_ = effect of *i*th BDS, *i* = 1 or 2; *C*_*j*_ = effect of *j*th carbohydrates additive, *j* = 1, 2, 3, or 4; (*SC*)_*ij*_ = interaction between *i*th BDS and *j*th carbohydrates additive; and *Ԑ*_*ijk*_ = random residual error.


$$\begin{array}{l} \;\;\;Y_{ijkl}=\mu +A_{i\;}+S_{i}+C_{jk}+{\left(SC\right)}_{ij}+\;\varepsilon_{ijkl}\;Model\;III \end{array}$$

where *Y*_*ijkl*_ = the observation, ruminal degradability of DM and N, ruminal kinetics; *µ* = overall mean common to all observations; *A*_*i*_ = fixed animal effect, *i* = 1, 2, or 3; *S*_*j*_ = effect of *j*th BDS, *j* = 1 or 2; *C*_*k*_ = effect of *k*th carbohydrate additive, *k* = 1, 2, 3, or 4; (*SC*)_*jk*_ = interaction between *j*th BDS and *k*th carbohydrates additive; and *Ԑ*_*ijkl*_ = random residual error.

Where significant differences between the treatment groups were detected, means were separated using the Tukey’s test (*α*= 0.05).

## RESULTS

### Chemical Composition of Fresh-Cut Napier Grass and 90-Day Silage

The nutrient composition of fresh-cut, pre-ensiled Napier grass is shown in Table 1. Fertilization with BDS increased (*P* < 0.05) pH and CP, and reduced (*P* < 0.05) fat content, with no effect (*P* > 0.05) on DM, WSC, Ash, NDF, ADF, and ADL (Table 2). Impacts of fertilization with BDS and carbohydrate additives at ensiling on Napier grass nutrient compositions are presented in Table 3. When forage was fertilized with BDS, molasses increased DM content of silage compared to the control and maize meal, but decreased fat content compared to no-fertilizer, maize meal silage, with significant (*P* < 0.05) interaction between the treatments for both nutrients. Fertilization with BDS did not affect (*P* > 0.05) the chemical composition of Napier silage. Additives increased (*P* < 0.01) silage DM content and reduced (*P* < 0.01) ADF and NDF content, with no effect (*P* > 0.05) on CP, NPN, or ADL content. Maize meal inclusion increased (*P* < 0.05) fat content while molasses inclusion increased (*P* < 0.01) the ash content. Carbohydrate additives and BDS treatment combinations had no effect (*P* > 0.05) on any silage fermentation characteristics (Table 3). While fertilization with BDS had no effect (*P* > 0.05) on silage fermentation characteristics, molasses increased WSC and decreased NH_3_-N content (*P* < 0.05) compared with the control and the maize meal treatment. The additives did not affect (*P* > 0.05) pH and LA content of the silage (Table 4).

**Table 2.** Dry matter (g kg^−1^), chemical composition (g kg^−1^ DM), and pH of fresh-cut Napier grass irrigated with and without bio-digester slurry

Parameters		Fertilization		SEM	Significance
	N	No slurry	Slurry		
DM	3	278.2	270.6	4.48	ns
pH	3	5.9^b^	6.0^a^	0.04	^*^
WSC	3	54.3	53.6	2.23	ns
CP	3	105.9^b^	118.6^a^	2.96	^*^
Ash	3	61.4	66.7	2.69	ns
Fat	3	23.1^a^	16.9^b^	1.15	^*^
NDF	3	788.2	790.6	6.41	ns
ADF	3	562.3	580.6	6.90	ns
ADL	3	28.4	28.6	13.50	ns

DM, dry matter; WSC, water soluble carbohydrate; CP, crude protein; NDF, neutral detergent fiber; ADF, acid detergent fiber; ADL, acid detergent lignin; g kg^−1^, grams per kilogram; g kg^−1^ DM, grams per kilogram dry matter; SEM, standard error mean.

Row means with different superscripts differ significantly at *P* < 0.05.

*P* < 0.05; ns, not significant: *P* > 0.05.

**Table 3.** Dry matter, chemical composition of Napier grass silage after 90 days of ensiling

Fertilization	Additives	*N*	DM	CP	NPN	Fat	Ash	NDF	ADF	ADL
			(g kg^−1^)	(g kg^−1^ DM)						
No slurry	No-additive	3	286.5^b^	78.5	9.8	17.3^ab^	81.0	759.2	500.1	73.9
	Molasses	3	342.9^ab^	100.6	9.3	16.1^ab^	106.7	583.0	368.8	65.0
	Maize meal	3	346.3^ab^	92.0	7.9	16.8^ab^	65.5	581.2	372.5	65.4
	Brown sugar	3	304.4^b^	89.8	8.2	20.1^ab^	66.3	678.5	418.0	66.9
Slurry	No-additive	3	275.6^b^	86.7	8.1	22.0^ab^	70.0	754.5	500.0	66.2
	Molasses	3	397.4^a^	107.0	8.9	12.9^b^	105.1	621.2	330.4	45.6
	Maize meal	3	316.1^b^	90.4	9.0	24.0^a^	64.4	607.6	389.5	52.6
	Brown sugar	3	336.9^ab^	94.2	6.0	17.3^ab^	58.2	648.1	398.0	58.2
SEM			14.89	7.89	1.05	1.93	5.08	31.82	27.30	31.91
Fertilization										
No slurry		12	320.0	90.2	8.8	17.6	80.0	650.5	414.8	67.8
Slurry		12	331.5	94.6	8.0	19.0	74.4	657.8	404.5	55.6
SEM			7.45	3.95	0.52	0.96	2.54	15.91	13.65	15.96
Additive										
	No-additive	6	281.0^c^	82.6	8.9	19.7^ab^	75.5^b^	756.9^a^	500.1^a^	70.0
	Molasses	6	370.1^a^	103.6	9.1	14.5^b^	105.9^a^	602.1^b^	350.0^b^	55.3
	Maize meal	6	331.2^ab^	91.2	8.4	20.4^a^	65.0^b^	594.4^b^	381.0^b^	59.0
	Brown sugar	6	320.7^bc^	92.0	7.1	18.7^ab^	62.3^b^	663.3^b^	408.0^b^	62.6
SEM			10.53	5.58	0.74	1.36	3.59	22.50	19.30	22.57
Significance										
	Fertilization (F)		ns	ns	ns	ns	ns	ns	ns	ns
	Additives (A)		^**^	ns	ns	^*^	^**^	^**^	^**^	ns
	F x A		^*^	ns	ns	^*^	ns	ns	ns	ns

DM, dry matter; CP, crude protein; NPN, non-protein nitrogen, NDF, neutral detergent fiber, ADF, acid detergent fiber; ADL, acid detergent lignin; *N*, number of observations; g kg^−1^, grams per kilogram; g kg^−1^ DM, grams per kilogram dry matter; SEM, standard error mean.

Column means with different superscripts differ significantly at *P* <0.05.

**P < 0.01; *P < 0.05; ns, not significant: *P* > 0.05.

**Table 4.** Fermentation characteristics of Napier grass silage after 90 days of ensiling

Fertilization	Additives	*N*	pH	Fermentative characteristics		
				WSC	LA	NH_3_-N
				(g kg^−1^ DM)		(g kg^−1^ TN)
No slurry	No-additive	3	4.4	14.0	30.2	45.9
	Molasses	3	4.2	29.4	29.9	14.8
	Maize meal	3	4.3	20.7	21.7	41.4
	Brown sugar	3	4.1	21.0	31.1	22.1
Slurry	No-additive	3	4.4	14.8	26.1	37.8
	Molasses	3	4.2	28.3	30.9	13.3
	Maize meal	3	4.5	15.6	16.8	46.4
	Brown sugar	3	3.9	22.2	29.5	15.9
SEM			0.27	3.91	6.66	12.03
Fertilization						
No slurry		12	4.3	21.3	28.2	31.0
Slurry		12	4.3	20.2	25.8	28.3
SEM			0.14	1.96	3.33	6.02
Additive						
	No-additive	6	4.4	14.4^b^	28.1	41.8^a^
	Molasses	6	4.2	28.9^a^	30.4	14.0^b^
	Maize meal	6	4.5	18.1^ab^	19.2	43.9^a^
	Brown sugar	6	4.0	21.6^ab^	30.3	19.0^ab^
SEM			0.19	2.77	4.71	8.51
Significance						
	Fertilization (F)		ns	ns	ns	ns
	Additives (A)		ns	^*^	ns	^*^
	F x A		ns	ns	ns	ns

WSC, water soluble carbohydrate; LA, lactic acid; NH_3_-N, Ammonium Nitrogen, *N*, number of observations; g kg^−1^ DM, grams per kilogram dry matter; g kg^−1^ TN, grams per kilogram total nitrogen; SEM, standard error mean.

Column means with different superscripts differ significantly at *P* < 0.05.

*P < 0.05; ns, not significant: *P* > 0.05.

### In Sacco DM and CP Degradability Kinetics

Mean degradability parameter values obtained by fitting the model of Ørskov and McDonald (1979), defining the kinetics of DM degradation and ED at three rumen fractional outflow rates, are presented in Table 5. There were interactions between BDS fertilization and additive inclusion (*P* < 0.05) in the silage on “*a*,” “*b*,” and “*a* + *b*”. Maize meal increased (*P* < 0.01) the soluble fraction “*a*” compared with the brown sugar in no fertilized treatments. Maize meal and molasses increased the fraction “*a*” (*P* < 0.01) compared to the control in BDS fertilized treatments. On the other hand, molasses increased the fraction “*b*” (*P* < 0.01) compared to the control and brown sugar in the no fertilization treatment. However, fertilization and additive inclusion did not affect the fraction “*b*” (*P* > 0.05). Silage from the no fertilization treatment containing no-additives had lowest (*P* < 0.01) PD “*a* + *b*”. However, there was no BDS × carbohydrate additive interaction (*P* > 0.05) on the degradation rate constant “*c*” and ED of Napier grass silage at 2%, 5%, and 8 % outflow rate. Fertilization with BDS increased (*P* < 0.05) soluble fraction “*a*,” slowly degradable fraction “*b*” and PD “*a* + *b*,” and ED at 2%, 5%, and 8% outflow rate, but had no effect (*P* > 0.05) on rate of degradation “*c*.” Carbohydrate additives increased (*P* < 0.01) the soluble fraction “*a*,” slowly degradable fraction “*b*” and PD “*a* + *b*,” and ED at 2%, 5%, and 8% outflow rate but had no effect (*P* > 0.05) on rate of degradation “*c*.”

**Table 5.** Degradability constants and calculated effective degradability at three passage rates for dry matter disappearance of Napier grass silage after 90 days of ensiling

Fertilization	Additives	*N*	Degradability constants (%)				ED (%) at different outflow rates		
			*a*	*b*	*c*	*a* + *b*	*k* = 0.02	*k* = 0.05	*k* = 0.08
No slurry	No-additive	6	18.0^abc^	55.2^c^	0.005	73.2^d^	55.4	44.2	38.1
	Molasses	6	16.9^abc^	80.3^a^	0.007	97.2^a^	68.3	52.9	44.6
	Maize meal	6	21.7^a^	74.4^ab^	0.008	96.1^ab^	69.1	54.9	47.3
	Brown sugar	6	16.1^bc^	65.7^b^	0.016	81.8^cd^	57.4	44.9	38.2
Slurry	No-additive	6	14.5^c^	71.9^ab^	0.008	86.4^bc^	60.3	46.6	39.2
	Molasses	6	21.5^a^	74.1^ab^	0.008	95.6^ab^	72.1	56.9	48.7
	Maize meal	6	20.4^ab^	73.5^ab^	0.004	93.9^ab^	67.5	53.3	45.7
	Brown sugar	6	19.6^abc^	73.0^ab^	0.010	92.6^ab^	65.9	52.0	44.5
SEM			1.10	2.28	0.0047	2.30	2.21	1.81	1.61
Fertilization									
No slurry		24	18.2	68.9^b^	0.009	87.1^b^	62.5^b^	49.2^b^	42.1^b^
Slurry		24	19.0	73.1^a^	0.008	92.1^a^	66.4^a^	52.2^a^	44.5^a^
SEM			0.55	1.14	0.0023	1.15	1.10	0.90	0.80
Additive									
	No-additive	12	16.3^b^	63.5^c^	0.007	79.8^c^	54.9^b^	45.4^b^	38.7^b^
	Molasses	12	19.2^ab^	77.2^a^	0.008	96.4^a^	70.2^a^	54.9^a^	46.6^a^
	Maize meal	12	21.0^a^	74.0^ab^	0.006	95.0^a^	68.3^a^	54.1^a^	47.3^a^
	Brown sugar	12	17.8^ab^	69.4^bc^	0.013	87.2^b^	61.6^b^	48.5^b^	41.4^b^
SEM			0.78	1.61	0.0033	1.62	1.56	1.28	1.14
Significance									
	Fertilization (F)		ns	^*^	ns	^**^	^*^	^*^	^*^
	Additives (A)		^**^	^**^	ns	^**^	^**^	^**^	^**^
	F x A		^**^	^**^	ns	^**^	Ns	Ns	ns

*a*, soluble fraction; *b*, insoluble but potentially degradable fraction; *a* + *b*, potential degradability; *c*, outflow rate of degradation (h^−1^); ED, effective degradability; *k*, rumen outflow rate (h^−1^); *N*, number of observations; %, percentage; SEM, standard error mean.

Column means with different superscripts differ significantly at *P* < 0.05.

**P < 0.01; *P < 0.05; ns, non-significant: *P* > 0.05.

Mean degradability parameters obtained by fitting the model of Ørskov and McDonald (1979) defining the kinetics of CP degradation and ED at three rumen fractional outflow rates, are presented in Table 6. There was no BDS fertilization × carbohydrate additive interaction (*P* > 0.05) on all CP degradation kinetics of the silages. Fertilization with BDS had no effect (*P* > 0.05) on all CP degradation kinetics of the silages. However, silage with molasses increased (*P* < 0.05) PD “*a* + *b*” compared to the control treatment.

**Table 6.** Degradability constants and calculated effective degradability at three passage rates for crude protein disappearance of Napier grass silage after 90 days of ensiling

Fertilization	Additives	N	Degradability constants (%)				ED (%) at different outflow rates		
			*a*	*b*	*c*	*a* + *b*	*k* = 0.02	*k* = 0.05	*k* = 0.08
No slurry	No-additive	6	7.8	56.9	0.007	64.8	45.2	33.3	26.9
	Molasses	6	11.9	68.2	0.002	80.1	56.7	42.8	35.3
	Maize meal	6	11.7	63.0	0.002	74.7	55.1	41.6	34.3
	Brown Sugar	6	10.7	60.8	0.002	71.6	52.3	39.6	32.6
Slurry	No-additive	6	7.0	58.1	0.001	65.1	46.9	34.9	28.5
	Molasses	6	15.5	70.3	0.001	85.8	64.7	49.8	41.7
	Maize meal	6	9.1	50.8	0.001	84.2	60.5	44.9	36.5
	Brown Sugar	6	11.7	58.7	0.002	70.3	52.1	39.5	32.8
SEM			4.79	9.33	0.0023	6.06	5.07	4.73	4.56
Fertilization									
No slurry		24	10.5	62.2	0.003	72.8	52.5	39.3	32.3
Slurry		24	10.8	59.5	0.002	76.4	56.1	42.3	34.8
SEM			2.39	4.66	0.0012	3.03	2.54	2.36	2.28
Additive									
	No-additive	12	7.4	57.5	0.004	64.9^b^	46.1	34.1	27.7
	Molasses	12	13.7	69.2	0.002	82.9^a^	60.7	46.3	38.5
	Maize meal	12	10.4	56.9	0.002	79.4^ab^	57.8	43.3	35.4
	Brown Sugar	12	11.2	59.8	0.002	71.0^ab^	52.5	39.6	32.7
SEM			3.39	6.60	0.0017	4.29	3.59	3.34	3.23
Significance									
	Fertilization (F)		ns	ns	ns	ns	Ns	ns	ns
	Additives (A)		ns	ns	ns	^*^	Ns	ns	ns
	F x A		ns	ns	ns	ns	Ns	ns	ns

*a*, soluble fraction; *b*, insoluble but potentially degradable fraction; *a* + *b*, potential degradability; *c*, outflow rate of degradation (h^−1^); ED, effective degradability; *k*, rumen outflow rate (h^−1^); *N*, number of observations; %, percentage; SEM, standard error mean.

Column means with different superscripts differ significantly at *P* < 0.05.

*P < 0.05; ns, non-significant: *P* > 0.05.

## DISCUSSION

### Chemical Composition of Fresh-Cut Napier Grass

In the present study, the DM content of fresh-cut Napier was within the range of 250–400 g kg^−1^ DM content which is considered optimal for satisfactory fermentation (Wilkinson, 2005). However, the observed DM content of fresh-cut Napier was lower compared to findings by Lubisi (2014), who reported DM content of 400 and 330 g kg^−1^ for BDS and no BDS fertilized Napier grass, respectively. The difference might be attributed to the stage of maturity at harvest, and different sampling procedures. The reduction of pH by fertilization with BDS contradicts findings by Lubisi (2014), who reported similar pH content of fresh-cut Napier grass fertilized with and without BDS. However, the pH content of fresh-cut Napier grass materials before ensiling was between 5 and 6, similar to that reported by Kung (2010).

Napier grass typically contains low levels of WSC (Nisa 2006; Bureenok et al., 2012). In the current study, the WSC concentration in Napier grass exceeded the minimum recommended concentration for effective fermentation of 37 g WSC kg^−1^ DM (Haigh, 1990). The findings of the present study are consistent with previous (Markos and Fulpagare, 2015) findings on *Pennisetum* grown in tropical environments.

The positive influence of BDS on CP contradicted findings by Lubisi (2014). In addition to the effect of the stage of maturity at harvest, and to different sampling procedures, the disparity could be attributed to differences in soil fertility, the quality of the BDS, and to climatic factors.

### Fermentation Characteristics and Chemical Composition of Napier Grass Silages

Silage DM in all treatments exceeded 300 g kg^−1^, which indicated good quality silage. Increased DM content during silage fermentation reduces the chances of Clostridial colonization (McDonald et al., 2011). High DM content of the silage in response to inclusion of molasses was consistent with Lubisi (2014)

The high residual WSC content in silage including carbohydrate additives was expected, with the highest residual WSC concentrations obtained with the molasses addition. Similar results were reported by Mtengeti et al. (2006). Higher residual WSC could be beneficial to ruminants, because of better palatability (Tava et al., 1995) and increased ruminal carbohydrate availability. In the current study, LA from maize meal treated silage remained low (19.2 g kg^−1^ DM) compared to molasses treated silage, which had the highest (30.9 g kg^−1^ DM) LA content. A range of 60–100 g LA kg^−1^ DM is desirable to retain DM and energy and preserve the silage for a long period of time. Therefore, the LA fermentation was sub-optimal in this study.

The substantial numerical reduction in the CP content of silages compared to the ensiled material across all treatments was similar to findings by Lubisi (2014). Less CP in ensiled forage may be due to protein hydrolysis coupled with proteolysis into soluble products such as free amino acids and NH_3_-N (Duniére et al., 2013) easily lost through slippage, which should be managerially minimized to retain the N for ruminal assimilation into microbial protein. Leibensperger and Pitt (1988) reported that when silage pH drops to 4.3 or lower, then silage proteolytic activity is reduced. In this study, all silages contained less than 100 g NH_3_-N kg^−1^ of total N, which was indicative of well-preserved silage (McDonald et al., 2011). However, protein degradation still occurred despite the rapid decrease in pH caused by the rapid production of inhibitory LA from the high levels WSC in all treatments. The high NH_3_-N particularly in maize meal-treated silage was thought to be caused by excessive protein breakdown resulting in a slower reduction in pH, which could allow Clostridia to penetrate the silage ecosystem (Kung, 2001), which may reduce silage palatability. There are no comparable studies on the effects of BDS treatment and additives on inhibiting protein degradation during ensiling. In the present study, NPN content of all silages was less than 120–150 g kg^−1^ typically associated with Clostridial colonization (Kung, 2010).

In the present study, the additives differentially affected the chemical composition of the silage. The greater effect of molasses on the ash content was supported by Gofen and Khalifa (2007) but was contradicted by the findings of Lubisi (2014). Gofen and Khalifa (2007) reported that molasses itself has high mineral content which contributes to the silage ash content when used as a carbohydrate additive. Molasses decreased fat content of the fertilized silage compared to maize in fertilized silage, which confirmed findings by Mokoboki et al. (2016). The decrease in additive-treated silage NDF and ADF content could be partially explained by a dilution effect of low fiber additives, and, given elevated N, by stimulation of insoluble fiber fermentation. Similar findings were reported by Lubisi (2014). Low NDF and ADF content in additive-treated silages are consistent with previous studies on Napier grass (Mtengeti et al., 2006; Bureenok et al., 2012; Lubisi, 2014). In contrast to the present findings, Zereu et al. (2015) reported reduced silage ADL contents due to additives. The disparity could be due to the different chemistry of the forage materials.

### In Sacco DM and CP Degradability Kinetics

The DM degradability of feeds is a key variable for evaluating the nutritive value of forages. Fertilization with BDS increased the DM degradability of silage, likely an effect of higher CP content and higher degradability of the additives, which factors suggest enhanced microbial degradation in the rumen. The effect of fertilizers on in sacco DM degradability of forage grasses is not well defined. The highly soluble and digestible sugars and the starch from the additives contributed to increased DM disappearance in the rumen (Gomes et al., 2015). Furthermore, these carbohydrates likely promoted fermentative degradation of cell wall non-starch polysaccharides (Nasehi et al., 2014) leading to improvement in the DM degradability. On the other hand, low DM degradability could be attributed to the high ADL content. While Gül et al. (2008) and Kaya et al. (2009) similar reported a higher ruminal DM degradability of grass silage after 8, 16, 24, and 48 h of incubation due to carbohydrate additives, Granzin and Dryden (2005) did not find such association.

Information regarding the effect of fertilizers on in sacco degradability of forage CP is lacking. In the present study, carbohydrate additives did not protect silage CP from ruminal degradation. Nowak et al. (2004) reported similar effects. A high rate of CP disappearance from the molasses treated Napier grass silage could favor high concentration of NH_3_ in the rumen. Lower CP degradability in the untreated silage could be due to high lignin content which acts as a mechanical barrier inhibiting microbial action (Van Soest, 1994).

### DM and CP Degradability Kinetics

Molasses treated silage had higher residual WSC content compared to the control treatment, which likely contributed to a greater fraction “*a*” of DM. In the present study, both fractions “*a*” and “*b*” for DM were lower than reported by Nowak et al. (2004). The higher DM PD (*a* + *b*) of BDS treated silage may be related to the protein content of fresh ensiled forage. Superior degradability of molasses treated silage is from the fraction “*b*,” consistent, which was consistent with the analyzed lignin content. The increase in PD of CP with additives suggests decreased quantity of CP entering the small intestine (Nowak et al., 2004) and would need supplement for high producing cattle.

## CONCLUSIONS

Napier silage quality depended on both BDS fertilization and the added fermentable carbohydrate substrates. Fertilization with BDS increased pH and CP, and reduced-fat content of fresh-cut Napier. Additives increased silage DM content, with reduced fiber (ADF, NDF) content. Relative to the control, molasses inclusion increased silage WSC, reduced NH_3_-N, while its combination with BDS treatment increased silage DM, with less fat compared to the BDS combination with maize meal inclusion. Measurement of butyric and other fatty acids could further clarify the stoichiometry the fermentation in relation to silage quality. For DM, the BDS fertilization with no silage additives had the least “*a*” fraction, while the no BDS, no additive silage had the least “*b*” fraction, with the least PD for the no BDS, no-additive silage treatment. At different outflow rates (*k* = 0.02, 0.05, 0.08), fertilization increased the ED of the DM, similar to the effect of molasses and maize meal inclusion. Relative to the control, molasses inclusion increased the PD of silage CP. Collectively, our results suggested that BDS fertilization of Napier grass with addition of readily fermentable carbohydrate substrate, particularly from molasses, induced changes in silage chemical and fermentation characteristics likely to promote better forage preservation and ruminal microbial digestion. This approach is especially appealing to farmers of lower economic means to improve their ability to produce ruminant animals efficiently.
